# Embedding patients' values and preferences in guideline development for allergic diseases: The case study of Allergic Rhinitis and its Impact on Asthma 2024

**DOI:** 10.1002/clt2.12377

**Published:** 2024-06-11

**Authors:** Rafael José Vieira, Bernardo Sousa‐Pinto, Antonio Bognanni, Juan José Yepes‐Nuñez, Yuan Zhang, Justyna Lityńska, Ewelina Sadowska, Ewa Borowiack, Boleslaw Samolinski, Alkis Togias, Torsten Zuberbier, Jean Bousquet, Holger J. Schünemann

**Affiliations:** ^1^ Department of Community Medicine Information and Health Decision Sciences (MEDCIDS) Faculty of Medicine University of Porto Porto Portugal; ^2^ Centre for Health Technology and Services Research Health Research Network (CINTESIS@RISE) Faculty of Medicine University of Porto Porto Portugal; ^3^ Department of Health Research Methods, Evidence and Impact McMaster University Hamilton Ontario Canada; ^4^ Department of Medicine Evidence in Allergy Group McMaster University Hamilton Ontario Canada; ^5^ School of Medicine Universidad de los Andes Bogotá Colombia; ^6^ Pulmonology Service Internal Medicine Section Fundación Santa Fe de Bogotá University Hospital Bogotá Colombia; ^7^ Evidence Prime Kraków Poland; ^8^ Department of Prevention of Environmental Hazards, Allergology and Immunology Medical University of Warsaw Warsaw Poland; ^9^ Division of Allergy, Immunology, and Transplantation (DAIT) National Institute of Allergy and Infectious Diseases NIH Bethesda Maryland USA; ^10^ Institute of Allergology Charité – Universitätsmedizin Berlin Corporate Member of Freie Universität Berlin and Humboldt‐Universität zu Berlin Berlin Germany; ^11^ Fraunhofer Institute for Translational Medicine and Pharmacology ITMP, Immunology and Allergology Berlin Germany; ^12^ Fraunhofer Cluster of Excellence for Immune‐Mediated Diseases CIMD Frankfurt Germany; ^13^ Michael G. DeGroote Cochrane Canada & McMaster GRADE Centres McMaster University Hamilton Ontario Canada; ^14^ Department of Medicine McMaster University Hamilton Ontario Canada; ^15^ Department of Biomedical Sciences Humanitas University Milan Italy

**Keywords:** allergic rhinitis, GRADE, guidelines, utility, values and preferences

## Abstract

Recommendations for or against the use of interventions need to consider both desirable and undesirable effects as well as patients' values and preferences (V&P). In the decision‐making context, patients' V&P represent the relative importance people place on the outcomes resulting from a decision. Therefore, the balance between desirable and undesirable effects from an intervention should depend not only on the difference between benefits and harms but also on the value that patients place on them. V&P are therefore one of the criteria to be considered when formulating guideline recommendations in the Evidence‐to‐Decision framework developed by the Grading of Recommendations, Assessment, Development and Evaluations (GRADE) Working Group. Patients' V&P may be quantified through utilities, which can be elicited using direct methods (e.g., standard gamble or time trade‐off) or indirect methods (using validated instruments to measure health‐related quality of life, such as EQ‐5D). The GRADE approach recommends conducting systematic reviews to summarise all the available evidence and assess the degree of certainty on V&P. In this article, we discuss the importance of considering patients' V&P and provide examples of how they are considered in the 2024 person‐centred Allergic Rhinitis and its Impact on Asthma (ARIA) guidelines.

## 1 INTRODUCTION

Healthcare interventions (e.g., pharmacological treatments, surgical procedures, policy interventions, etc.) are associated with outcomes, leading to desirable or undesirable health effects. For example, an oral H_1_‐antihistamine for the treatment of allergic rhinitis (AR) may lead to reduced nasal symptoms (desirable health effect) but also to an increased risk of side effects such as somnolence (undesirable health effect).^1^ Therefore, formulating a recommendation on an intervention requires considering both desirable and undesirable effects, something for which patients' values and preferences (V&P) are essential.

In the decision‐making context, V&P represent the relative importance people place on the outcomes of interest that result from a decision or intervention.^2^ Considering the previous example, this would correspond to the importance that patients attribute to the possibility of having their nasal symptoms improved over the risk of having side effects. Therefore, V&P, in the guideline development process, do not deal with patients' preferences regarding different treatments (nor with intervention costs, cultural differences or acceptability, although these elements are also relevant for the formulation of recommendations) but rather with the outcomes associated with the interventions. This distinction is crucial because the evaluation of the balance between desirable and undesirable effects (or benefits and harms) from an intervention depends on (i) the risk difference resulting from an intervention (i.e., the difference between benefits and harms) and (ii) the value patients place on the benefits and harms. Thus, while V&P may not directly address patients' treatment preferences, they do concern patients' preferences across health states resulting from treatments.

Patients' V&P are one of the criteria to account for when formulating recommendations of interventions according to the Grading of Recommendations, Assessment, Development and Evaluation (GRADE) Evidence to Decision Framework (EtD) (used by over 100 organisations).^3^, ^4^, ^5^, ^6^

In this article, we will consider the example of AR (in the frame of the Allergic Rhinitis and its Impact on Asthma (ARIA) guidelines) to describe (i) the importance of assessing V&P to inform health recommendations and (ii) how to assess and synthesise evidence on such V&P.

## 2 WHY DO VALUES INFLUENCE THE BALANCE OF BENEFITS AND HARMS?

Decisions in health care require evidence about the outcomes prevented or caused by interventions as well as knowledge on their relative importance to the population of interest (that is, the weight that a population assigns to these outcomes). Such knowledge is essential for guideline panels to determine the balance of benefits and harms. For example, considering that a second‐generation oral H_1_‐antihistamine for the treatment of AR leads to a 20% absolute reduction in nasal symptoms and a 20% absolute increase in mild side effects:

- If nasal symptoms are judged equally important as mild side effects, the balance of benefits and harms neither favours nor disfavours the use of the oral H_1_‐antihistamine.
- If nasal symptoms are judged more important than mild side effects, the balance of benefits and harms favours the use of the oral H_1_‐antihistamine.

While V&P might vary when considering individual patients, panels need to have information on V&P at a group or societal level for formulating guideline recommendations, as guidelines are made for the average patient. However, if there is any substantial uncertainty or variability regarding the V&P at a group/societal level, decisions strongly depend on an individual's V&P and guideline developers may prefer to make conditional recommendations.

## 3 HOW CAN V&P BE ASSESSED?

The most common way of quantitatively measuring V&P is through the so‐called ‘utilities’. Utilities are numerical representations of the value assigned to different outcomes. They provide an index measure anchored on a scale, with 1 reflecting ‘perfect health’ and 0 reflecting ‘being dead’.^2^, ^7^ Several methods may elicit the utilities associated with outcomes, including direct and indirect approaches.^8^, ^9^

Direct approaches of measuring utilities include the standard gamble, time trade‐off and rating scales.^2^, ^8^, ^10^ Both the standard gamble and the time trade‐off assess the relative importance patients place on outcomes by putting them in a situation of uncertainty. Rating scales involves asking individuals to rate the desirability or value of a health state on a numerical visual analogue scale, which can then be converted into a utility value.^2^, ^8^, ^10^

Indirect methods use validated instruments to measure health‐related quality of life. They allow us to define health profiles converted into utilities using country‐specific value sets.^11^ An example is the EQ‐5D questionnaire, which assesses health‐related quality of life across five dimensions (mobility, self‐care, usual activities, pain/discomfort and anxiety/depression). Based on patients' responses to these dimensions, it is possible to estimate a utility value through validated value sets.^12^, ^13^ This has been done for AR and asthma.^14^ Importantly, not all quality‐of‐life instruments allow for the direct assessment of utility values. For example, the Rhinitis Quality of Life Questionnaire measures quality‐of‐life in patients with AR but does not define health profiles that may be directly converted into utilities.^15^, ^16^

## 4 HOW ARE V&P USED WHEN MAKING GUIDELINE RECOMMENDATIONS?

In the GRADE approach, V&P are one of the criteria of EtD.^3^, ^4^, ^5^, ^6^, ^17^ This framework helps to formulate guideline recommendations based on the best available evidence across 12 criteria and is used globally. As with other criteria, the GRADE approach aims to summarise the available evidence and assess the degree of certainty on V&P, if possible, using information from systematic reviews.^18^, ^19^ This allows for decisions to be aligned with V&P. Although the systematic assessment of V&P is an emerging field, several examples show how to conduct such an assessment and inform guideline panel members.^20^, ^21^

Without high certainty evidence on V&P, guideline panels may generate their evidence by administering outcome utility rating surveys to broader groups, such as patient advocacy groups or, more commonly, panel members (including patient representatives and clinical experts). These outcome utility surveys may use a visual analogue scale for panel members to rate the health utility of outcomes.^22^, ^23^, ^24^, ^25^ In this context, ensuring a common understanding of the health outcomes by all guideline panel members is crucial. This can be achieved through health outcome descriptors—that is, explicit and detailed definitions of all outcomes concerning symptoms, time horizon, testing and treatment, and consequences written in a patient‐friendly way and based on the experiences of the affected individuals or patients^23^, ^24^, ^26^ (Box 1). Beyond serving the purpose of rating outcome utility, outcome descriptors are relevant for outcome prioritisation and for additional steps in the EtD framework, including communication with the users of recommendations on which outcomes were considered.^23^, ^24^, ^26^

**Box 1 Example of health outcome descriptor for ‘Nasal symptoms’ in intermittent allergic rhinitis.**

1 **Nasal symptoms**

*Population: Intermittent Allergic Rhinitis*

**Symptoms**

You experience nasal symptoms. These symptoms may include a runny nose, which is when your nose is running with clear, watery discharge. You may also have nasal congestion when your nose feels stuffy and blocked. You could also have nasal itching, which may make you want to rub or scratch your nose. Sneezing is also a common symptom, and you may find yourself sneezing more often than usual. The more severe your symptoms are, the more you will be bothered by them.

**Time Horizon**

Your symptoms can come and go. You will experience nasal symptoms less than 4 days a week or less than 4 weeks in a row.

**Testing and Treatment**

You can often manage your nasal symptoms with over‐the‐counter or prescription medications. These medications may be taken intranasally or orally. If your symptoms are severe, a healthcare provider may recommend immunotherapy, which may be given orally or by injection to help reduce your allergic reactions over time. Nasal symptoms may often be assessed using patient diaries, which reflect the past 12 or 24 h.

**Consequences**

Nasal symptoms may be bothersome and affect your daily life. They may lead to sleep disturbances, decreased work and school productivity, and reduced overall quality of life. However, with proper management and treatment, your nasal symptoms can often be controlled, allowing you to enjoy a better quality of life and relief from the discomfort they cause.

## 5 INCORPORATING V&P RELATED TO AR IN GUIDELINE DEVELOPMENT: THE EXAMPLE OF ARIA

The ARIA initiative^27^, ^28^ has long recognised the need for person‐centred guidelines. Therefore, for the next revision of the ARIA guidelines (ARIA 2024), we conducted a systematic review to synthesise and appraise all available evidence on patients' V&P for health outcomes associated with AR.^29^

Our systematic review of 36 primary studies found that:

- Increased AR severity or comorbid asthma were associated with lower utilities, therefore having a larger impact than that of considering whether AR was seasonal or perennial.
- Patients consistently rate nasal symptoms, particularly nasal congestion, as the most important and bothersome symptoms, followed by ocular and non‐nasal respiratory symptoms.
- Patients value symptom relief more than the risk of side effects from medications.

Even though the certainty of the evidence tended to be generally low in our systematic review, and some of the primary studies had important limitations (prompting the need for unbiased future utility studies assessing larger cohorts of patients and making use of real‐world data), decision‐making in guideline development must be based on the best available evidence.^30^, ^31^ Accordingly, while the ARIA 2020 guideline (developed from ARIA 2016) already tried to have a person‐centred approach, the 2024 update will use the findings of this systematic review to make patients' V&P explicit and inform panel members while going through the EtD framework.^32^, ^33^ For example, given our findings that patients value symptom relief over side effect concerns, if an oral H_1_‐antihistamine provides 20% symptom relief but carries a 20% risk of mild side effects, panel members may choose to recommend the oral H_1_‐antihistamine. This highlights the practical relevance of our systematic review in guiding clinical decisions as well as the importance of tailoring recommendations to patients' V&P. Additionally, ARIA 2024 will use health outcome descriptors to ensure (i) a common understanding by all panel members regarding the assessed outcomes and (ii) that outcomes are defined based on the experiences of AR patients. Finally, ARIA 2024 will integrate direct patient data collected through mHealth tools such as MASK‐air^®^ into the decision‐making process.^34^, ^35^, ^36^ This is part of the effort to include evidence more closely related to patients' reality (referred to by some as ‘real world evidence’, despite certain concerns with this expression^37^), thereby expanding the evidence base.

## 6 CONCLUSION

Patients' V&P are critical for decision‐making, particularly for guideline panel members to determine the balance of health benefits and harms. Systematic reviews focused on patients' V&P are an emerging and critical field that can provide panel members with the best available evidence.

## AUTHOR CONTRIBUTIONS

**Rafael José Vieira**: Writing – original draft; writing – review & editing. **Bernardo Sousa‐Pinto**: Writing – original draft; writing – review & editing. **Antonio Bognanni**: Writing – original draft; writing – review & editing. **Juan José Yepes‐Nuñez**: Writing – original draft; writing – review & editing. **Yuan Zhang**: Writing – original draft; writing – review & editing. **Justyna Lityńska**: Writing – original draft; writing – review & editing. **Ewelina Sadowska**: Writing – original draft; writing – review & editing. **Ewa Borowiack**: Writing – original draft; writing – review & editing. **Boleslaw Samolinski**: Writing – original draft; writing – review & editing. **Alkis Togias**: Writing – original draft; writing – review & editing. **Torsten Zuberbier**: Writing – original draft; writing – review & editing. **Jean Bousquet**: Writing – original draft; writing – review & editing. **Holger J. Schünemann**: Writing – original draft; writing – review & editing.

## CONFLICT OF INTEREST STATEMENT

The authors declare no conflicts of interest.

## Data Availability

Data sharing is not applicable to this article as no new data were created or analysed in this study.
